# Early-Onset Retinal Dysfunction Associated with Novel WDR19 Variants in Sensenbrenner Syndrome

**DOI:** 10.3390/diagnostics15131706

**Published:** 2025-07-03

**Authors:** Bogumiła Wójcik-Niklewska, Zofia Oliwa, Zofia Zdort, Adrian Smędowski

**Affiliations:** 1 Department of Pediatric Ophthalmology, Faculty of Medical Sciences in Katowice, Medical University of Silesia, 40-055 Katowice, Poland; asmedowski@sum.edu.pl; 2 Professor Kornel Gibiński University Hospital Center, Medical University of Silesia, 40-514 Katowice, Poland; 3 Students’ Scientific Society, Department of Ophthalmology, Faculty of Medical Sciences in Katowice, Medical University of Silesia, 40-752 Katowice, Poland; zocha2002@gmail.com (Z.O.); zosia.zdort@gmail.com (Z.Z.); 4 GlaucoTech Co., 40-282 Katowice, Poland

**Keywords:** cranioectodermal dysplasia, *WDR19* mutations, retinal dystrophy, ciliopathy, children

## Abstract

*Sensenbrenner syndrome*, or cranioectodermal dysplasia (CED), is a rare autosomal recessive ciliopathy characterized by craniofacial, skeletal, ectodermal, and renal abnormalities. Ocular involvement, though infrequent, can include retinal dystrophy with early-onset visual impairment. We report a case of a 2-year-old boy with classic clinical features of CED and significant ocular findings. Genetic testing revealed two novel compound heterozygous variants in the *WDR19* gene—c.1778G>T and c.3536T>G—expanding the known mutational spectrum associated with this condition. Ophthalmologic evaluation demonstrated bilateral optic nerve hypoplasia, high hyperopia, and severely reduced ERG responses, consistent with global retinal dysfunction. Fundoscopy revealed optic disk pallor, vessel attenuation, and peripheral pigment changes. Multisystem findings included postaxial polydactyly, brachydactyly, short stature, hypotonia, and stage 2 chronic kidney disease. This case highlights the importance of early ophthalmologic screening in suspected CED and underscores the utility of ERG in detecting early retinal involvement. The identification of two previously undescribed *WDR19* variants contributes to genotype–phenotype correlation in CED and emphasizes the need for ongoing documentation to guide diagnosis, management, and genetic counseling.

**Figure 1:**
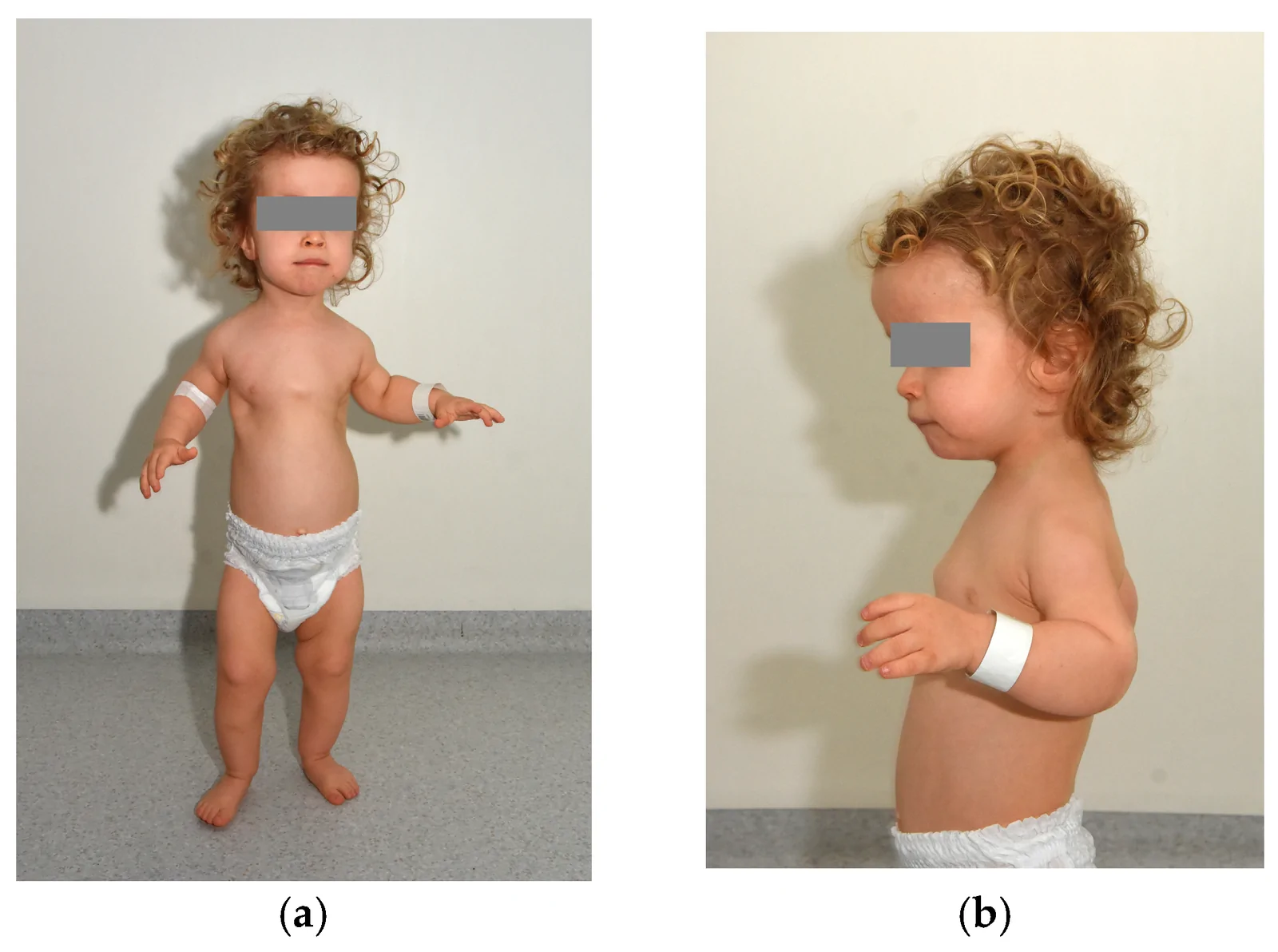
We present the case of a 2-year-old boy diagnosed with cranioectodermal dysplasia (*Sensenbrenner syndrome*). Genetic testing revealed two novel, previously unreported variants in the *WDR19* gene—c.1778G>T (inherited from his mother) and c.3536T>G (inherited from his father). Further tests (aCGH and FGFR3 tests) did not reveal any other abnormalities. Prenatal findings included shortening of the long bones, postaxial polydactyly of the left hand, and a frontal soft-tissue edema. The child was delivered by cesarean section at 39 weeks of gestation, with a birth weight of 3750 g. Immediately after birth, shortening of the long bones and polydactyly were confirmed, and hypotonia was observed. Laboratory findings indicated significant renal insufficiency, and visual impairment was also suspected. Panels (**a**,**b**) depict frontal and lateral views of the patient, demonstrating phenotypic features characteristic of *Sensenbrenner syndrome* (cranioectodermal dysplasia). Craniofacial dysmorphisms include a prominent high forehead and a square face, a small nose with a relatively deep nasal root and retrognathia. A short neck, a narrow thorax with short ribs, a distended and flaccid abdomen, diastasis recti, bilateral inguinal hernias, a left hydrocele, postaxial polydactyly of the left hand, brachydactyly of both hands, shortening of the long bones, syndactyly of the 2nd–3rd toes on the right foot and generalized hypotonia are also seen. *Sensenbrenner syndrome*, also referred to as cranioectodermal dysplasia (CED), is a rare ciliopathy characterized by multisystem involvement, including distinctive skeletal, ectodermal, and craniofacial anomalies [1]. Typical skeletal manifestations include a narrow thoracic cage, shortening of the proximal limbs, and digital anomalies such as polydactyly, syndactyly, and brachydactyly [1]. Ectodermal features encompass sparse scalp hair, skin laxity, nail dysplasia, and dental anomalies such as hypodontia and widely spaced, underdeveloped teeth [2]. Craniofacial dysmorphisms are frequently observed, such as a prominent forehead, low-set ears, epicanthal folds, telecanthus, full cheeks, and an everted lower lip [3]. *Sensenbrenner syndrome* is a part of a broader group of disorders known as ciliopathies, which are caused by dysfunctions in cilia—microtubule-based organelles present in nearly all human cells. These structures serve as critical signaling hubs that regulate key developmental pathways and maintain tissue homeostasis. A key mechanism underlying ciliogenesis and ciliary signaling is intraflagellar transport (IFT), which facilitates the bidirectional movement of molecular cargo along the ciliary axoneme [4]. Anterograde transport, from the ciliary base to the tip, is mediated by the IFT-B complex in conjunction with the kinesin-2 motor protein, whereas retrograde transport, from the ciliary tip back to the base, is driven by the IFT-A complex coordinated with the dynein-2 motor [5,6]. Most pathogenic variants identified in individuals with CED involve genes encoding components of the IFT-A complex, including *IFT122*, *WDR35* (*IFT121*), *WDR19* (*IFT144*), *IFT43*, and *IFT140*. Reported mutations in *WDR19* comprise missense variants such as L710S (OMIM: 608151.0001) and R1178Q (OMIM: 608151.0010), the nonsense mutation R1103X (OMIM: 608151.0002), a frameshift mutation resulting from a single base-pair deletion (OMIM: 608151.0016), and a splice-site mutation (OMIM: 608151.0017) [3,7]. These mutations are predominantly inherited in an autosomal recessive manner, with affected patients harboring biallelic pathogenic variants, either as compound heterozygotes or homozygotes [3]. The clinical findings and the two pathogenic *WDR19* variants confirmed the diagnosis of *Sensenbrenner syndrome*. The child requires multidisciplinary care, including regular ophthalmologic and nephrological follow-up and comprehensive physical rehabilitation. He was admitted to the pediatric ophthalmology ward for detailed evaluation of his visual impairment.

**Figure 2:**
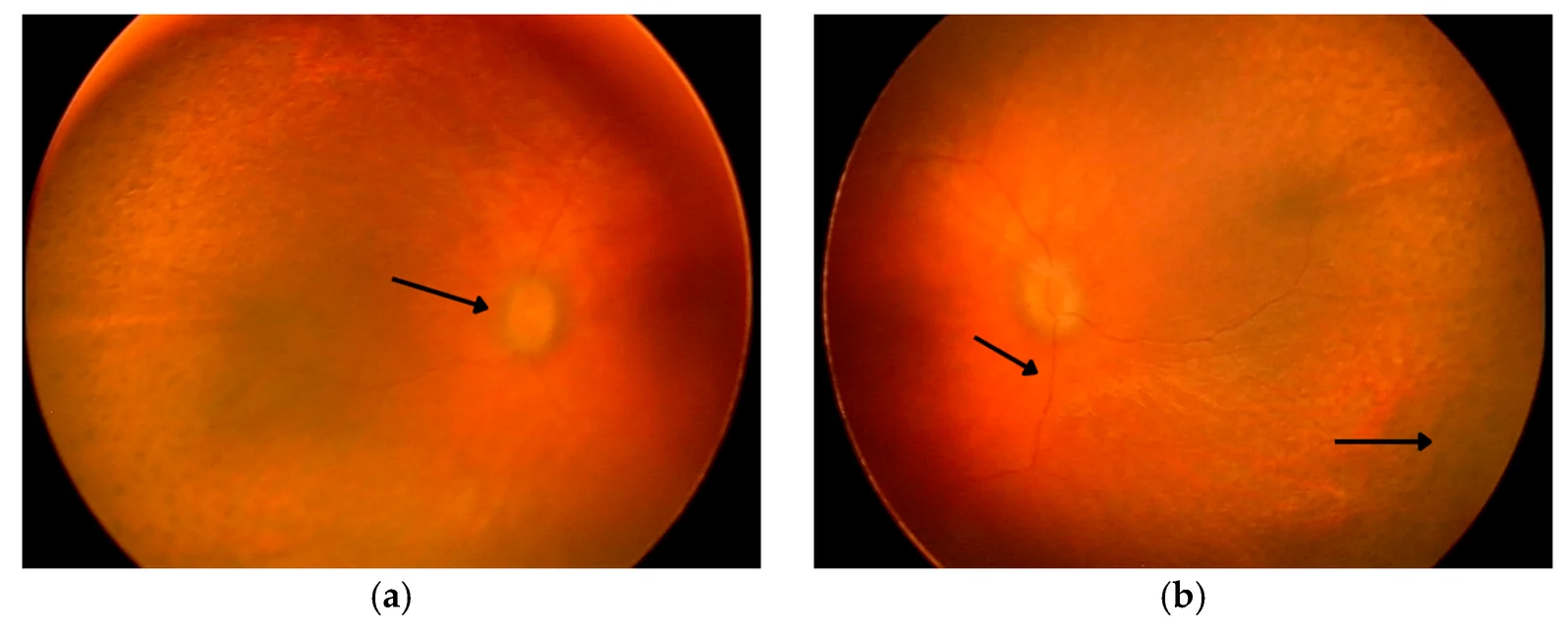
Panels (**a**,**b**) present fundus photographs of the patient’s left and right eye obtained under general anesthesia. The first arrow indicates the optic disc, the second points to markedly attenuated retinal vessels, and the third highlights areas of pigment rearrangement. Both optic disks appear pale pink with blurred margins and a filled-in physiological cup. The macula lacks a foveal reflex, and the retinal vessels are markedly attenuated. Peripheral retina exhibits multiple areas of pigment rearrangement, resembling a leopard-spot pattern.

**Figure 3:**
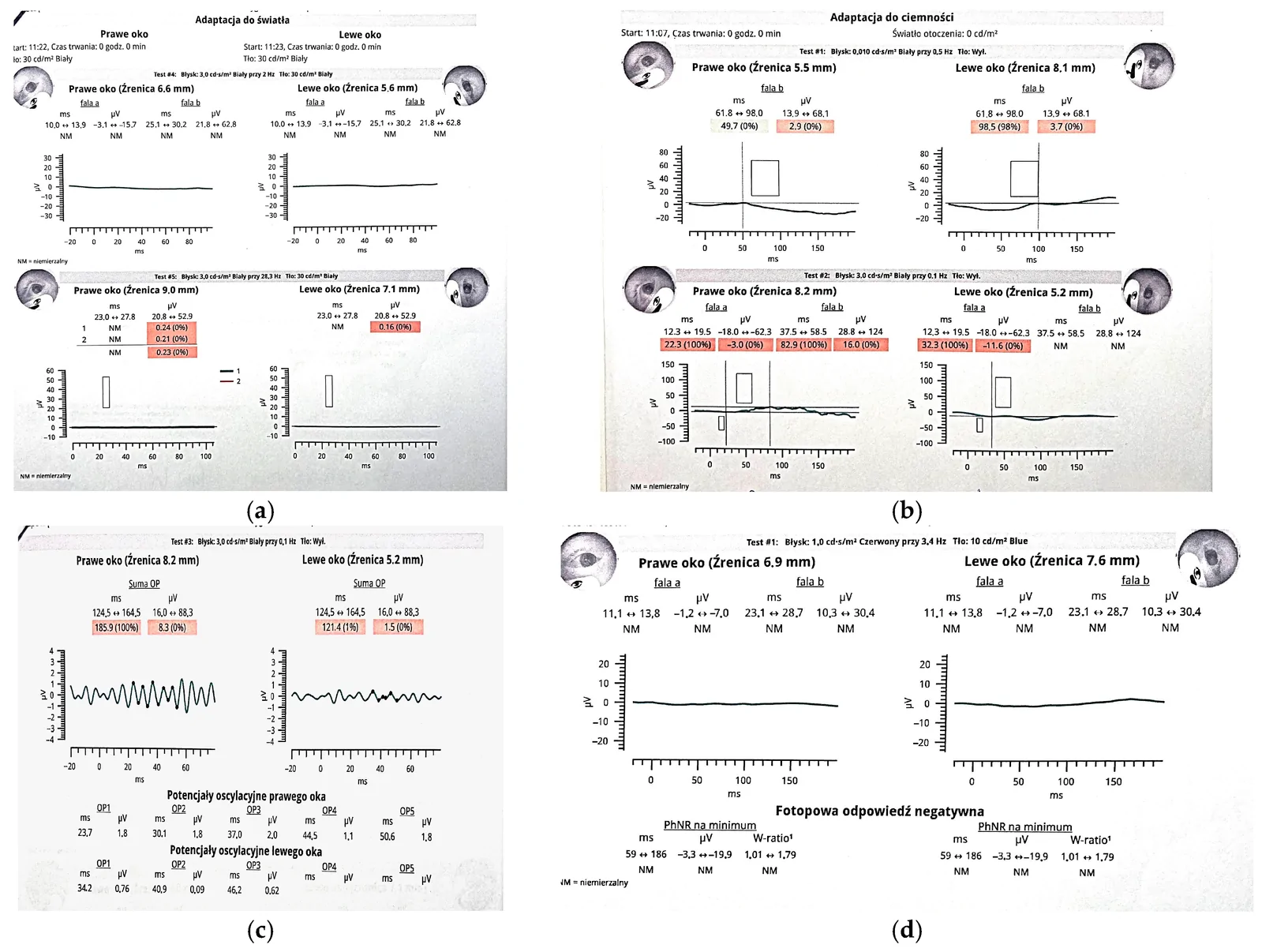
Panels (**a**–**d**) present RETeval^®^ testing results. (**a**) Photopic ERG: cone responses are either unmeasurable (“NM”) or below 0.3 µV, consistent with complete cone dysfunction. (**b**) Scotopic ERG: b-wave amplitudes in both eyes fall below the 5th percentile, with prolonged latencies, indicating severely reduced rod function and secondary bipolar cell dysfunction. (**c**) Oscillatory Potentials (OPs): summed OP amplitudes are 8.3 µV (right eye) and 1.5 µV (left eye), both at the 0th percentile, reflecting near-complete loss of amacrine cell activity. (**d**) Photopic Negative Response (PhNR): minimal amplitude and unmeasurable W-ratio in both eyes, suggesting significant ganglion cell impairment. This ERG profile supports a global retinal dysfunction affecting both outer and inner retinal layers. Although ocular involvement has been documented in cranioectodermal dysplasia, it is not universally seen. Retinal dystrophy affects around a quarter of patients, but when present, symptoms often include nyctalopia with early childhood onset [2]. In overlapping ciliopathies like Bardet–Biedl syndrome, progressive retinal degeneration typically results in severe visual impairment by young adulthood, and a similar clinical trajectory may be expected in CED [2]. Electroretinographic (ERG) abnormalities can be detected in children as young as four years, with both scotopic and photopic responses showing significant reduction or extinction [2]. Fundoscopic findings may reveal hallmarks of retinal dystrophy such as attenuation of retinal vessels and peripheral bone-spicule-shaped pigmentary deposits [7], typically observed between ages five and eleven. Other ocular findings in CED include refractive errors such as hyperopia, myopia, and astigmatism, as well as strabismus (e.g., esotropia) and euryblepharon [2]. Given the potential for early-onset visual decline, ophthalmologic screening should be initiated by the age of four. However, if vision loss or nyctalopia is suspected, ERG and fundoscopy should be performed at an earlier age. Annual follow-up is essential to monitor disease progression. The early introduction of low-vision aids and specialized educational resources can significantly enhance functional vision and support developmental needs [2]. The combination of skeletal, ectodermal, craniofacial, and retinal findings in our patient largely resembles the few pediatric cases of *Sensenbrenner syndrome* described to date; however, the two novel *WDR19* variants we report expand the so far recognized genetic spectrum of this ciliopathy. We believe that reporting patients with CED is crucial, not only to clarify genotype-phenotype correlations and the natural history of early-onset visual decline, but also to refine evidence-based guidelines for multidisciplinary monitoring, intervention and family counseling in this rare disorder.

## Data Availability

The original contributions presented in this study are included in the article. Further inquiries can be directed to the corresponding authors.
